# Radiofrequency Catheter Ablation Of Atrioventricular Nodal Reentry Tachycardia In A Patient With Inferior Vena Cava Anomaly

**Published:** 2009-07-01

**Authors:** Murugesan Karthigesan, Shenthar Jayaprakash

**Affiliations:** Sri Jayadeva Institute of Cardiology, Bangalore, India

**Keywords:** Left inferior vena cava, hemiazygos vein, atrioventricular nodal reentrant tachycardia

## Abstract

Curative radiofrequency catheter modification of the slow pathway is the recommended therapy for patients suffering from recurrent symptomatic atrioventricular nodal reentry tachycardia. This is usually performed via femoral vein and the inferior vena cava (IVC). Presence of venous occlusion or complex venous anomaly involving the IVC may preclude this approach. Here, we report a case with a complex venous anomaly involving the inferior vena cava, who underwent electrophysiological study and successful radiofrequency ablation by an alternative approach.

## Introduction

Atrioventricular nodal reentry tachycardia (AVNRT) is the most common form of paroxysmal supraventricular tachycardia in adults [1]. Radiofrequency catheter ablation of slow atrioventricular nodal pathway is an effective treatment for AVNRT which is usually done through inferior vena cava approach [2]. Anatomically inferior vena cava is formed by the union of right and left common iliac veins on the right anterior surface of fifth lumbar vertebra. Entire transposition of the inferior vena cava to the left, with hemiazygos continuation draining into the coronary sinus via left superior vena cava is an extremely rare anomaly in an otherwise structurally normal heart and situs. We report a patient with this anomaly who underwent electrophysiological study and subsequently successful radiofrequency ablation for AVNRT using a superior vena cava approach.

## Case report

A 55-year-old female diabetic and hypertensive presented with a history of recurrent episodes of paroxysmal palpitations since six years despite oral verapamil. Physical examination was unremarkable. ECG during tachycardia showed narrow QRS complexes at the rate of 200 beats/min with ECG in sinus rhythm being normal. Echocardiogram was otherwise normal except dilated coronary sinus. In view of recurrent supraventricular tachycardia, she was taken up for electrophysiology study and radiofrequency catheter ablation.

**Figure 1:**
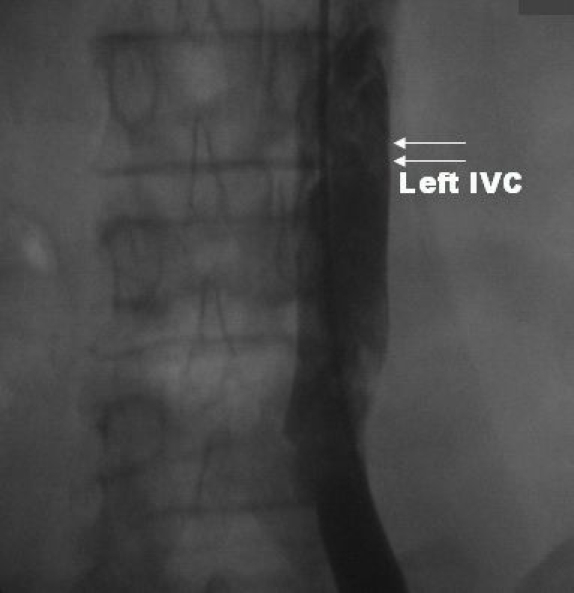
Fluoroscopy in the posterior-anterior view with contrast injection from left femoral vein shows Inferior vena cava (IVC) on the left side of the spine indicating a Left sided IVC.

**Figure 2:**
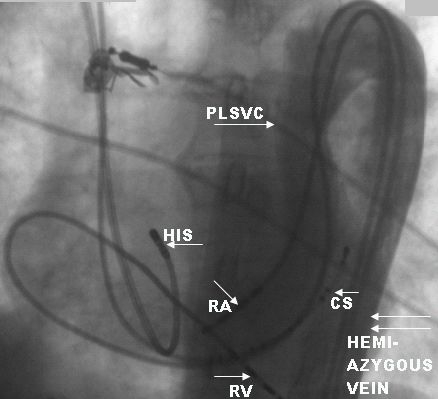
Fluoroscopy in the LAO view shows hemiazygos venous continuation to persistent LSVC and draining to coronary sinus. PLSVC- Persistent left superior vena cava, CS-coronary sinus catheter, HIS- HIS bundle catheter, RA- right atrial catheter, RV- right ventricle catheter

Initially a 6F decapolar catheter (2-5-2 spacing) was positioned in the coronary sinus (CS) through right internal jugular vein. The left femoral vein was cannulated and a 6F quadripolar catheter was advanced under fluoroscopy guidance. As the catheter was advanced, it was noticed to be on left side of the spine. Hence 20 ml of non-ionic contrast medium was injected through the vascular introducer sheath. This revealed the presence of inferior vena cava on the left side of the spine (Figure 1), which continued as dilated hemiazygos vein, which in turn drained to dilated coronary sinus through a persistent left superior vena cava (Figure 2). Subsequently a 6F quadripolar (5mm spacing) catheter was advanced through anomalous venous channel and could be manipulated to the RV apex. But a second quadripolar catheter could only be advanced to low RA which was left in place for programmed electrical stimulation as stimulation from the CS catheter failed to capture the atrium due to large CS. Since the third catheter could not be manipulated, right internal jugular vein was cannulated and a 7F deflectable quadripolar catheter was used because of its stability to record the His bundle electrogram (Figure 2).

**Figure 3:**
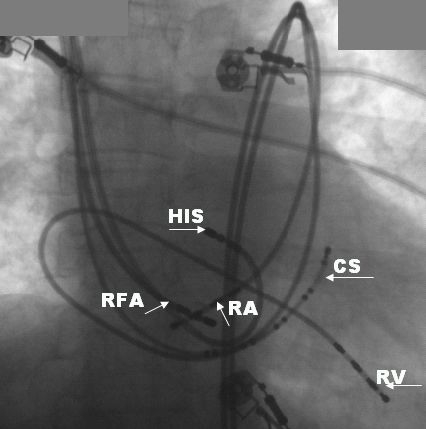
Fluoroscopy in the RAO view shows recording and ablation catheter positions. CS-coronary sinus catheter, HIS- HIS bundle catheter, RA- right atrial catheter, RV- right ventricle catheter, RFA- radiofrequency ablation catheter

During EP Study patient was found to have a dual AV nodal physiology. A narrow QRS tachycardia was easily induced with programmed electrical stimulation with tachycardia cycle length of 280ms and an activation sequence similar to AV node reentry tachycardia. We performed third right internal jugular vein puncture to introduce the ablation catheter. The tip of the catheter was positioned at the posterior atrial septum just above the coronary sinus ostium (Figure 3). The ablation electrode was stabilized at a target site that was associated with an AV ratio of 1:4 to 1:8. Radiofrequency current was delivered at maximum power of 40W and a maximum temperature of 60ºC for a maximum duration of 30 seconds each. Delivery of RF energy was associated with an accelerated junctional rhythm. A total of eight RF applications were applied. There was no inducible SVT after the procedure with programmed stimulation without and with isoproterenol infusion and the procedure was uncomplicated. Ultrasonography of the abdomen was done before discharge showed normal visceral situs, direct drainage of hepatic veins into the RA and the presence of left sided IVC ascending posterior to the aorta. No recurrences of tachycardia were noted at six months follow up.

## Discussion

Presence of persistent left superior vena cava (PLSVC) is considered to be one of the most frequently encountered anomalies of the systemic venous return. Estimated prevalence of the anomalies of inferior vena cava (IVC) in the general population is about 0.07%–8.7% [3]. Left-sided IVC occurs in 0.2-0.5% of the general population and in most cases it crosses over to the right side via the left renal vein or more inferior to that and crossover is usually anterior but rarely posterior to the aorta [4]. Entire transposition of the IVC to the left with hemiazygos continuation to PLSVC is extremely rare [5]. The incidence of this anomaly in an otherwise structurally normal heart and normal visceral situs is unknown.

Access to the right side chambers in the presence of this venous anomaly can be made either from femoral vein through an anomalous venous channel or through alternative venous accesses like basilic vein, subclavian vein or internal jugular vein. In the presence of anomalies of the IVC, positioning and manipulation of the recording catheters in right heart chambers is difficult from the femoral route, especially the RV and His bundle recording catheters. This form of venous anomaly also reduces the number of catheters that can be placed and manipulation of the catheter from the inferior route. Slow pathway modification with an ablation catheter is almost not possible. Conventional inferior vena cava approach for slow pathway ablation is difficult in complex congenital venous anomalies involving the inferior vena cava and contraindicated in the presence of IVC thrombus.

An alternative venous approach for the ablation via right subclavian or right internal jugular vein has been described [6-9]. Salem et al [9] reported a case series of slow pathway ablation via right internal jugular vein approach in patients with IVC filter. In the internal jugular approach, the shaft of the ablation catheter lies superior to the Eustachian ridge when the tip of the catheter is at the posterior right atrial septum. This eliminates the potential difficulties caused by the Eustachian ridge in positioning the ablation catheter tip at the target site in conventional femoral vein approach, which could be an advantage. The major disadvantage is greater radiation exposure to the operator due to a close proximity of the image intensifier to the operator and inability to use the side lead shield. Manipulating the catheter and viewing fluoroscopic images and intracardiac electrograms from an unconventional angle may also be difficult. The number of RF applications required for successful ablation was also relatively high in their case series. Successful slow pathway ablation via subclavian approach has also been described [6-8]. However, catheter stability in the slow pathway region is inadequate and fast pathway ablation was required for successful elimination of tachycardia by subclavian approach [7]. Successful ablation of right side accessory pathway by superior vena cava approach has been described in cases where conventional inferior vena cava approach had failed [10]. Successful ablation of typical atrial flutter by internal jugular approach has been described in a case of IVC anomaly [11]. We preferred internal jugular approach in the present case based on our previous experience with difficult right side accessory pathway ablation by this approach.

It is uncommon to place more than one vascular sheath in the internal jugular vein. We performed three internal jugular venous punctures in the present case based on our previous experience in performing two internal jugular venous punctures in young adults. Present case demonstrates the safety of placement of three separate 7F vascular introducer sheaths in the right internal jugular vein to accommodate one ablation and two recording catheters in adult patients. This case also demonstrate the feasibility of doing an electrophysiological study and RF ablation without compromising on the number of recording catheters even in the presence of complex venous anomaly involving IVC. In the present case, smaller size CS catheter could have been used and His bundle recording catheter could have been placed through right subclavian vein.  To the best of our knowledge this case is the first report of the use of the internal jugular vein for slow pathway ablation in complex venous anomaly.

## Conclusion

Abnormalities of the IVC are relatively uncommon. But it is an important condition that may be encountered by electrophysiologist. Catheter ablation of the slow AV nodal pathway can be performed safely and successfully by internal jugular approach in patients with complex venous anomaly involving inferior vena cava.

## References

[R1] Josephson ME (1978). Paroxysmal supraventricular tachycardia: an electro physiologic approach. Am J Cardiol.

[R2] Jackman WM (1992). Treatment of supraventricular tachycardia due to atrioventricular nodal reentry, by radiofrequency catheter ablation of slow pathway conduction. N Engl J Med.

[R3] Obernosterer A (2002). Anomalies of the inferior vena cava in patients with iliac venous thrombosis. Ann Intern Med.

[R4] Hirsh D (1963). Bilateral inferior vena cava. JAMA.

[R5] Brickner ME (1990). Left-sided inferior vena cava draining into the coronary sinus via persistent left superior vena cava: case report and review of the literature. Cathet Cardiovasc Diagn.

[R6] Avella A (2001). Radio frequency catheter ablation of atrioventricular nodal reentry tachycardia. Ital Heart J.

[R7] Machado C (2003). Radiofrequency catheter ablation of fast pathway via unconventional right subclavian venous access for atrioventricular reentrant tachycardia. J. Interv Cardiol.

[R8] Chandan K (2002). Slow pathway ablation without femoral access. Indian Heart J.

[R9] Salem YS (2006). Slow pathway ablation for atrioventricular nodal reentry using a right internal jugular vein approach: a case series. Pacing Clin Electrophysiol.

[R10] Yang HT (2001). Radiofrequency catheter ablation of right atrioventricular accessory pathway by superior vena cava approach. Hunan Yi Ke Da Xue Xue Bao.

[R11] Pai RK (2005). Radiofrequency catheter ablation of typical atrial flutter and the atrioventricular junction via the superior vena caval approach in a patient with a congenital absence of an inferior vena cava. J Interv Card Electrophysiol.

